# Comparative Analysis of Mechanical Properties and Microbiological Resistance of Polyfilament and Monofilament Suture Materials Used in the Operation “Tooth Extraction”

**DOI:** 10.3390/biomimetics8010129

**Published:** 2023-03-22

**Authors:** Alexey A. Pcheliakov, Ekaterina Yu. Diachkova, Yuriy L. Vasil’ev, Oxana A. Svitich, Alexander V. Poddubikov, Stanislav A. Evlashin, Beatrice A. Volel, Anastasia A. Bakhmet, Svetlana V. Klochkova, Ellina V. Velichko, Natalia Tiunova, Svetlana V. Tarasenko

**Affiliations:** 1 Department of Oral Surgery of Borovsky Institute of Dentistry, I.M. Sechenov First Moscow State Medical University (Sechenov University), Trubetskaya St. Bldg. 8\2, 119435 Moscow, Russia; 2 Department of Operative Surgery and Topographic Anatomy, I.M. Sechenov First Moscow State Medical University (Sechenov University), Trubetskaya St. Bldg. 8\2, 119435 Moscow, Russia; 3 Dentistry Faculty, Kazan State Medical University, 49 Butlerova Street St., 420012 Kazan, Russia; 4 I.I. Mechnikov Research Institute for Vaccines and Sera, 105064 Moscow, Russia; 5 Microbiology, Virology and Immunology Department, I.M. Sechenov First Moscow State Medical University (Sechenov University), Trubetskaya St. Bldg. 8\2, 119435 Moscow, Russia; 6 Skolkovo Institute of Science and Technology, 121205 Moscow, Russia; 7 Sklifosovskyi Institute of Clinical Medicine, I.M. Sechenov First Moscow State Medical University, St. Trubetskaya, 8, Bld. 2, 119991 Moscow, Russia; 8 Department of Human Anatomy and Histology, I.M. Sechenov First Moscow State Medical University (Sechenov University), Trubetskaya St. Bldg. 8\2, 119435 Moscow, Russia; 9 Department of Human Anatomy, Peoples Friendship University of Russia (RUDN University), Miklukho-Maklaya Str., 8, 117198 Moscow, Russia; 10 Department of Dental Diseases Propaedeutics, Peoples Friendship University of Russia (RUDN University), Miklukho-Maklaya Str., 8, 117198 Moscow, Russia; 11 Department of Medical Elementology, RUDN University, Miklukho-Maklaya Str., 8, 117198 Moscow, Russia; 12 Institute of Clinical Medicine, Department of Clinical Dentistry, National Research Lobachevsky State University of Nizhny Novgorod (Lobachevsky University, UNN), Pr. Gagarina 23, 603022 Nizhny Novgorod, Russia

**Keywords:** suture material, oral cavity microbiology, polyfilament, monofilament, physical properties, capillarity, extensibility, knot force

## Abstract

In surgical dentistry, suture material is the only foreign body that remains in the tissues after surgery, and it can lead to several negative reactions, for example, infection of the wound. The purpose of this study was to compare the mechanical properties and microbiological resistance of mono- and polyfilament suture materials used in tooth extraction operations. The study of elongation and knot force was carried out on an Instron 5969 Dual Column Testing System device. The capillarity of the materials was studied on a setup assembled by the authors manually by immersing the ends of the filaments in a colored manganese solution. A microbiological study was carried out on the threads taken for the experiment immediately after wound suturing, and on day 7, at which time they were removed. The comparison was made according to Rothia mucilaginosa, Streptococcus sanguinis, Staphylococcus epidermidis. Results: monofilament suture materials (Prolene and Glycolon), after calculating the Kruskal–Wallis and Mann–Whitney indices, showed better performance in all experiments compared to polyfilament sutures (Vicryl and PGA). In capillarity comparison, there was a significant difference between groups (*p* = 0.00018). According to the sum of the results of three microbiological studies on day 7, monofilament suture materials absorbed less of the studied bacteria on their surface compared to the polyfilament ones (*p* < 0.05). Conclusions: Of the studied suture materials, Prolene had the best microbiological resistance and good mechanical properties.

## 1. Introduction

When carrying out many surgical interventions, an important stage is the closure of the surgical field by connecting the tissues with suture materials [1]. In oral surgery, the suture material is one of the foreign bodies that remain in the tissues after manipulation and can lead to several negative reactions [2]. Threads can absorb microorganisms on their surface, allowing them to expand near the wound area after surgery [3]. With the advent of molecular genetic research methods, the study of the biology of microorganisms began as one of the leading triggers for the development and progression of inflammatory diseases of the dental system [4]. The occurrence of these infections is influenced by many factors: the presence of concomitant diseases, the ASA index, the grade of the surgical wound, the NNIS risk index. Important factors include the quality of the suture material, and its mechanical and chemical properties [5,6].

In modern surgical dentistry, new suture materials are being developed and existing ones are being improved [7], alongside the improvement of methods for their application [8]. According to some authors, postoperative complications with unknown etiology may be the result of improper suturing technique, resulting in untying of knots and loss of flaps’ tension [9].

All surgical sutures on the market are divided into monofilament and polyfilament sutures, both of which have both advantages and disadvantages.

Polyfilament suture material is characterized, in comparison with analogues from the group of traditional suture materials, by significant advantages; it is stronger, causes an insignificant tissue reaction, is strictly defined, and is close to optimal levels of strength loss and resorption. Dexon and Vicryl lose up to 80% of their strength in 2 weeks, and Polysorb in 3 weeks; they completely resolve within 2–3 months after surgery. However, this group of threads also has disadvantages; these are mainly related to their structure, which retains wick-capillary properties and the “sawing” effect. For example, to reduce the “saw effect”, Vicryl is coated with calcium stearate (coated Vicryl). As a result of this modification, the traumatic effect is reduced and the capillary properties of the thread are improved, with a simultaneous decrease in the strength of the knot, which requires the imposition of additional 2–3 knots for greater reliability.

Monofilament suture material is characterized, in comparison with analogues from the group of traditional suture materials, by significant advantages; due to the smooth surface of the monofilament, it has a weak traumatic effect when pulled. The threads retain high strength in tissues for a long time, losing only 30% of their strength in the first month. Most researchers note the following disadvantages of monofilament absorbable surgical threads: low strength (compared to polyfilament threads), significant loss of strength in the knot (if polypropylene loses 8–15% strength in the knot, then monofilaments lose 40–50%), low reliability of the knot (for strong tying of a monofilament, at least six knots are recommended) [1,5].

The purpose of this study is to compare the mechanical properties and microbiological resistance of mono- and polyfilament suture materials used in tooth extraction operations, Vicryl, Prolene, Glycolon, PGA Resorba, by studying them as a favorable microbiological environment for pathogenic and conditionally pathogenic aerobic microorganisms of dentition. These suture materials were selected as research materials based on the results of a survey of dentists based on I.M. Sechenov FMSMU.

## 2. Materials and Methods

**Figure 1:**
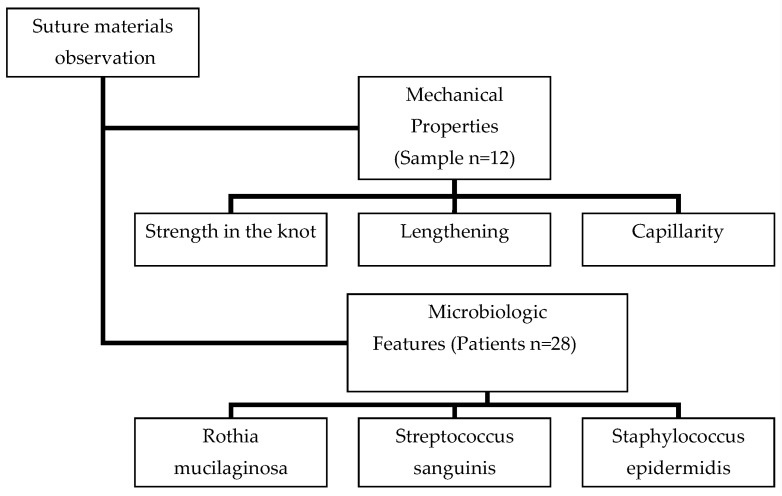
Study scheme.

The study of the materials considered in this article was performed in two stages (Figure 1—Scheme).

### 2.1. Study of Mechanical Properties

In an experimental study of the physical properties of synthetic suture materials used in dentistry, the following results were found in Table 1.

#### 2.1.1. Absorption Properties

The absorption properties of each suture material were studied on the basis of Skolkovo Institute of Science and Technology (Skoltech, Moscow, 121205, Russia). The setup for the experiment was assembled independently by the authors of this article and by Skoltech employees. The sample size was calculated using the formulas “Sample size” based on the results of a similar previously published study, and included three samples of each item (total *n* = 12 in the study) at a power of 80% and α = 5%.

The experimental setup consisted of two parts. The first one was a stand on which the threads under study were fixed with a special vise. The second is a tank with an 10% aqueous solution of KMnO_4_ (Renewal, JSC “PFC Renewal” 633621, Novosibirsk region, Suzunsky district, R. P. Suzun, Komissar Zyatkov str., 18), into which the ends of suture materials with weights attached to them were lowered to level out the shape memory effect in monofilament materials (Prolene and Glycolon).

The whole unit was hermetically sealed from the environment with a transparent plastic waterproof bag. The materials remained intact, without the influence of external mechanical forces on them, for 3 days. After the materials were removed from container, the stained part was measured with a ruler, cut, and measured one more time with a caliper.

#### 2.1.2. Tensile Strength at the Knot and Elasticity of the Material

**Figure 2:**
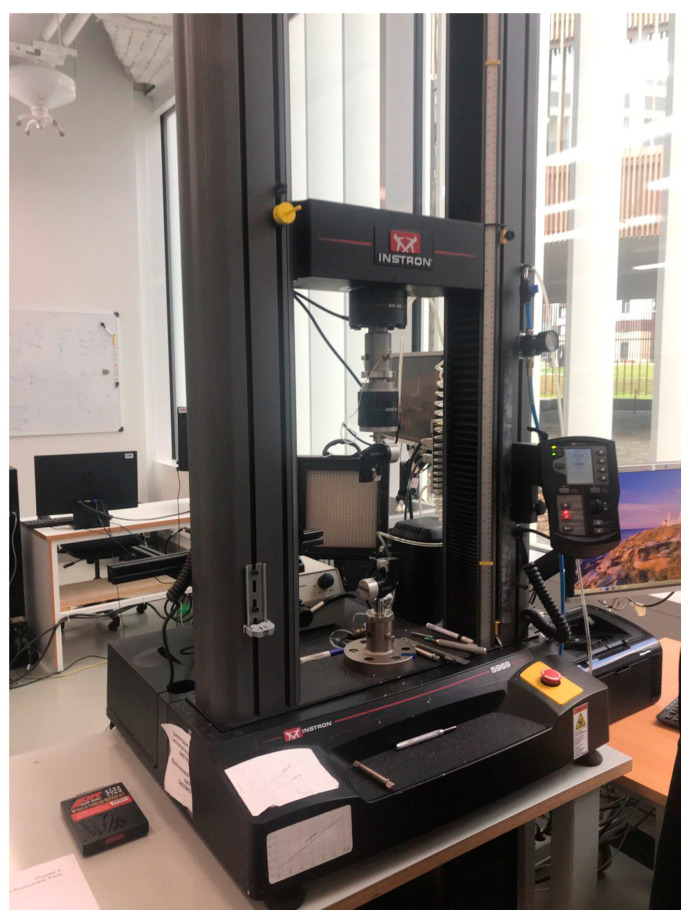
Instron 5969 Dual Column Testing System with material Glycolon in vise.

In the second experiment at Skoltech, properties such as knot strength and material elasticity were studied. These manipulations were performed using an Instron 5969 Dual Tensile Testing Machine Column Testing System (Figure 2), manufactured by Instron (Instron A Division Of Illinois Tool Works Inc.USA, Norwood). The knot under study, tied to each suture used in the study, is a simple 2-1-1 knot.

Vicryl, Prolene, Glycolon, PGA Resorba in this study formed two groups of 12 samples each (six samples of each name) of mono- and polyfilament suture materials (Table 1). The sample size was calculated using the formula’s sample size based on the results of a similar previously published study [9] (80% power, α = 5%). There were 24 samples in total.

Each surgical thread was divided into two parts, then tied back with a simple knot. The final length of each instance was 20 cm.

The samples were fixed in the vise of an Instron 5969 Dual Tensile Testing Machine Column Testing System. The working area between the vise of the machine was 170 mm, which was returned to by the machine method after each experiment. The knot on the samples was equidistant from the fixing elements.

The rate of rupture of the suture after launch was 100 mm/min, while data on the elongation and magnitude of the applied force from were recorded and transmitted from the Instron Sensors Series 2714 Cord and Yarn Grips.

### 2.2. Microbiological Examination

In a pilot clinical study of synthetic suture materials as a favorable microbiological environment for pathogenic and conditionally pathogenic aerobic microorganisms of the dentition, the same materials, Vicryl, Prolene, Glycolon, PGA Resorba, were used.

The pilot study involved adult patients with indications for tooth extraction who did not have common somatic diseases and who gave oral and written informed consent to participate in the study (Local Ethical Committee Protocol No. 22-21 dated 12/09/2021). The study was performed according to the Declaration of Helsinki on ‘‘Ethical Principles for the Conduct of Medical Research Involving Human Subjects’’ adopted by the World Medical Association (WMA) in 2016.

The following criteria were used for inclusion of patients in the study:

1. Availability of informed written consent of the patient to participate in the study
2. The age of the patient is from 18 to 70 years
3. Male and female patients
4. Indication for extraction of the teeth of the upper and lower jaws
5. Absence of severe somatic diseases

The following criteria were used for exclusion of patients from the study:

1. Age less than 18 years
2. Pregnancy, breastfeeding
3. The presence of concomitant diseases in the stage of exacerbation and decompensation
4. Lack of indications for tooth extraction

The following criteria were used for early discontinuation exclusion of patients from the study:

1. Refusal of the patient of further participation in the study
2. Exacerbation of concomitant pathologies
3. Failure to comply with medical recommendations

In the study of suture materials as a favorable microbiological environment for pathogenic and conditionally pathogenic aerobic microorganisms of the dental system, seven samples of each material (*n* = 28–28 patients, each with one tooth extracted, 28 extracted teeth) were studied in accordance with their name. The age of the included patients was 18–56 years (min: 18, max: 53. Median: 35.93), gender—men and women (12/16 patients, 1:1.3).

After an atraumatic extraction of the tooth, the socket was sutured with a suture of the chosen ‘‘envelope’’ technique, the Laurell–Gottlow suture. Immediately after the end of suturing, a section of the thread, located immediately after the needle, was cut off to 30 mm long and placed in a test tube with a sterile Tiga transport medium (manufacturer ‘‘Research Institute of Vaccines and Serums named after I.I. Mechnikov’’, Moscow). The test tube was frozen for transportation to the Federal State Budgetary Scientific Institution “I.I. Mechnikov Research Institute of Vaccines and Serums”. The microorganisms were defrosted and seeded on the day of sampling.

The material with bacteria absorbed on it was defrosted and seeded on meat peptone agar with the addition of 0.2% glucose and 5% blood [10]. A standard bacteriological examination was carried out in three stages:

1. Seeding of the test material in nutrient media

The study of pathological material began with microscopy. Microscopy of the stained native material made it possible to establish the approximate composition of the microbial landscape of the surgical thread.

With a sufficient content of pathogenic microorganisms in the sample, inoculation was carried out on dense culture medium (to obtain isolated colonies). The culture medium was chosen according to the requirements of the microorganisms.

The cultivation of microorganisms took place under parameters that exclude contamination (accidental contamination by foreign microbes) of the material under study. All utensils and culture medium were sterilized and, after inoculation of microbial material, protected from outside contamination. Manipulations with the test material were carried out in the flame zone of an alcohol lamp to exclude contamination of the material from the external environment, as well as to comply with safety regulations.

Inoculations of the material on culture medium were made no later than a day from the moment of their sampling and freezing.

2. Isolation of a pure culture

After a day of incubation, colonies grew on the plates. A colony is defined as a collection of microbes of the same species that have grown from a single microorganism. Since the material was a mixture of microbes, several types of colonies grew. Different colonies were marked with a pencil, outlined with a circle from the side of the bottom, and studied after. First, the colonies were studied with the naked eye: macroscopic signs were assessed. The cup was viewed (without opening it) from the bottom in transmitted light, the transparency of the colonies was noted (transparent, if it does not block light; translucent, if it partially blocks light; opaque, if light does not pass through the colony), and the size of the colonies was then measured (in mm). Then, the colonies were examined with magnification. To do this, a closed cup was placed upside down on an object table, the condenser was slightly lowered using a small magnification of the objective (×8), and the cup was moved. Microscopic features were studied in the colonies, including the nature of the edge and structure. Next, we studied the morphology of microbial cells from the colonies. To do this, smears were made from a part of each of the marked colonies with Gram staining. During the taking of colonies, the consistency was studied. When viewing smears, it was determined whether the colony was represented by one type of microbe in order to isolate a pure culture of bacteria. For this purpose, reseeding was carried out from the studied colonies onto a slant agar. The tubes were signed and incubated in a thermostat at 37 °C for 24 h.

3. Identification of microorganisms (determination of belonging to a species).

The last step was to determine the cultures absorbed on the suture material and their quantity; for their determination, a CFU/smear system was used.

On day 7, the suture material was removed from the area of the extracted tooth and placed in full in a test tube with Tiga transport medium. The subsequent stages of bacteriological research are completely duplicated with the steps described above.

The calculations and conclusions in this study were carried out on Rothia mucilaginosa, Streptococcus sanguinis and Staphylococcus epidermidis.

### 2.3. Statistical Processing of the Received Data

After checking the normality of the distribution of the sample using the Shapiro–Wilks method, non-parametric criteria were used for data processing (Kruskal–Wallis and Mann–Whitney) to compare all four groups and in pairwise comparison (mono-/polyfilament threads). When the differences between the samples were small or varied within a random area, the rank sums did not differ significantly. In this case, *p* > 0.05, which allowed us to say that no differences between groups were found. The results were considered statistically significant if the significance was greater than 95% (*p* < 0.05).

All work on statistical analysis was carried out in the programming language in the R program (v. 4.2.2 Hornik and R Core Team 2022).

Statistical data processing also included performing calculations of average values, and searching for the minimum and maximum, standard deviations, and medians in each group.

## 3. Results

### 3.1. Results of the Study of Mechanical Properties

The results of the study of physical properties were calculated and transferred to a tabular format (Table 2).

#### 3.1.1. Absorption Properties

In the first experiment, surgical sutures were divided into two groups, mono- (Prolene and Glycolon) and polyfilament (PGA and Vicryl) materials, due to the significant difference in the results in millimeters between them. Due to the inequality of the standard deviations of the results, the Mann–Whitney criterion was used to identify the difference between the groups. In study No. 1, monofilament materials (Prolene and Glycolon) were better than polyfilament materials (Vicryl, PGA Resorba) (*p* < 0.05) (Table 2).

**Figure 3:**
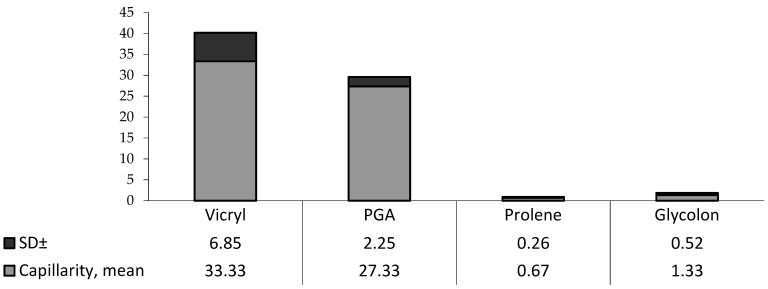
Diagram of the capillarity of each material in mm (mean ± SD).

To visualize the results, the median of each study is plotted on the chart (Figure 3).

#### 3.1.2. Tensile Strength at the Knot and Elasticity of the Material

Due to the inequality of the standard deviations of the results obtained in the experiments determining the knot force and the extensibility of materials, the Kruskal–Wallis criterion (*p* < 0.05) was used to identify the difference between groups in each study (*p* < 0.05) (Table 2.).

Calculation of the criterion according to the knot force values was carried out as follows.

According to the results of the calculations, there was a difference between the groups. Pairwise comparison of all four groups using the Mann–Whitney criterion and deciphering U from the critical value table found the following (*p* < 0.05):

− Vicryl had the highest knot force compared to PGA and Glycolon.
− Glycolon had the lowest knot force.

**Figure 4:**
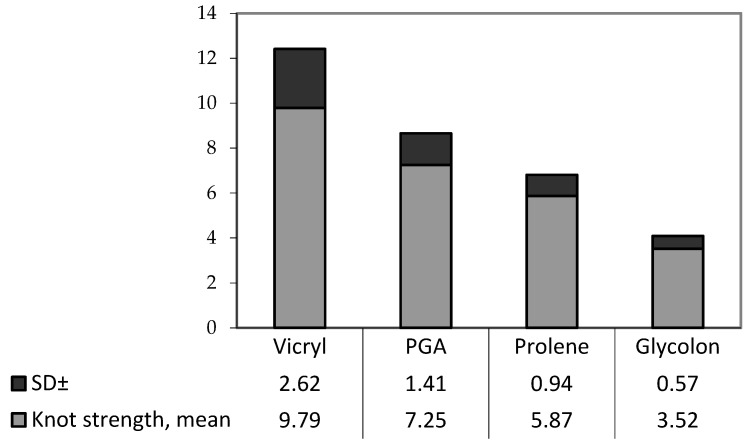
Diagram of the knot strength of each material in Newtons (mean ± SD).

To visualize the results, the median of each study is plotted on the chart. (Figure 4).

Calculations were also carried out to compare the differences between the two groups (monofilament and polyfilament suture materials) according to the Mann–Whitney criterion, and the following was concluded:

− Monofilament sutures of Prolene and Glycolon have lower knot strength compared to polyfilament sutures of Vicryl and PGA Resorba (*p* < 0.05).

Calculation of the criterion according to the values of extensibility found the following.

According to the results of the calculations, there was a difference between the groups. Pairwise comparison of all four groups using the Mann–Whitney criterion and deciphering U from the critical value table found (*p* < 0.05):

− Vicryl had the lowest extensibility compared to PGA, Prolene and Glycolon.
− Glycolon had the highest extensibility.

**Figure 5:**
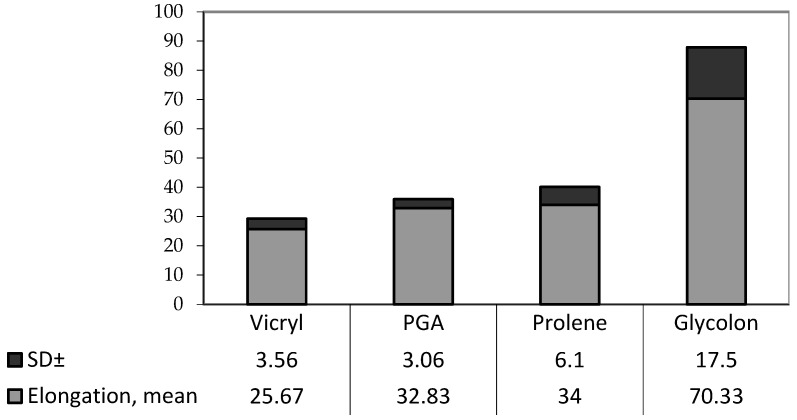
Diagram of the elongation of each material in mm (mean ± SD).

To visualize these results, the median of each study is plotted on the chart (Figure 5).

Calculations were also carried out to compare the differences in extensibility between the two groups (monofilament and polyfilament suture materials) according to the Mann–Whitney criterion.

### 3.2. Microbiological Investigation

The first stage of the experiment was to study the number of bacteria that are fixed on the suture material during the operation. This criterion is important, as the settled bacteria are brought into the tissues [11]. The results of the study of the first samples are presented in the table (Table 3).

Due to the inequality of the standard deviations of the results obtained in this experiment, the Kruskal–Wallis criterion was used to identify the difference between the groups.

**Figure 6:**
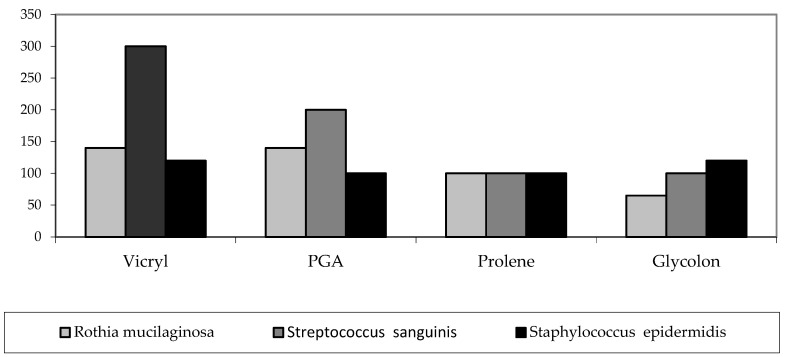
Diagram of the microbes’ volume for each material in the microbiological examination of threads during surgery (median).

To visualize the results, the median of each study is plotted on the chart (Figure 6).

Calculation by Rothia mucilaginosa:

The difference between groups in intraoperative samples were not statistically significant (*p* > 0.05) (Table 3).

However, when comparing mono- and polyfilament sutures using the Mann–Whitney criterion, less microbial absorption was found for monofilament materials (*p* < 0.05).

Calculation by Streptococcus sanguinis:

According to the results of calculations, there was a difference between the groups (*p* < 0.02) (Table 3). With the help of pairwise comparison of all four groups according to the Mann–Whitney criterion and decoding U according to the table of critical values, it was established that

− Most of the Rothia bacteria mucilaginosa was found on Vicryl samples (*p* < 0.05) compared to PGA, Prolene and Glycolon.
− Glycolon and Prolene showed lower bacterial contamination than Vicryl and PGA (monofilament sutures predominate over polyfilament sutures) (*p* < 0.05).

Calculation for Staphylococcus epidermidis:

There was no statistically significant difference between the groups (*p* > 0.05) (Table 3).

Due to differences in the structure of materials, the results of microbiological examination of threads taken on day 7 are more differentiated (Table 4).

**Figure 7:**
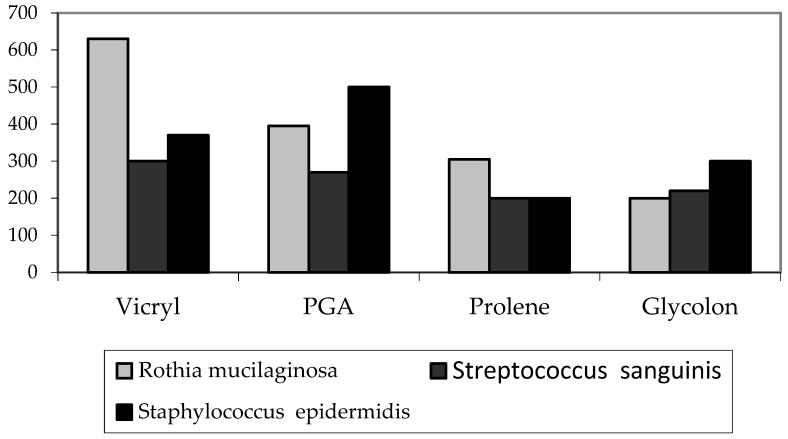
Diagram of microbes’ volume for each material in the microbiological examination of the threads on day 7 (median).

To visualize the results, the median of each study is plotted on the chart. (Figure 7).

At calculation of the Kruskal–Wallis criterion according to bacteria Rothia mucilaginosa, there was a significant difference between the groups. According to a U-test, (*p* < 0.05):

− Vicryl was the most inoculating material in comparison with PGA, Prolene and Glycolon.
− Within the group of monofilament materials, no difference was found
− PGA had more bacteria on its surface than Prolene and Glycolon.

In the study of Streptococcus sanguinis, differences were observed only between groups of mono- and polyfilaments (*p* < 0.05) (Table 4).

In the study of Staphylococcus epidermidis, statistically significant differences were observed, with predominant contamination by Vicryl (Table 4).

According to the Mann–Whitney U-criterion:

− No differences were found between Vicryl and PGA (*p* > 0.05).
− The best in terms of Staphylococcus epidermidis turned out to be Prolene (*p* < 0.05).

## 4. Discussion

Based on the results of the study of the mechanical properties of sutures, it was found that Vicryl has the highest force in the knot, due to which it better holds the wound edges in comparison with the other sutures considered. However, this material also has high wicking, due to which representatives of the microflora can penetrate deep into the tissues, with the risk of causing infection. Low elongation with a high knot force may indicate the formation of microcracks on the surface of the material because of the load, which means the material becomes even more susceptible to bacterial absorption.

− PGA was average in all three experiments.
− Glycolon and Prolene, as representatives of monofilament threads, had the lowest capillarity, which means the lowest probability of infection of surrounding tissues. With a high extensibility, followed by a weakening of the tension of the wound edges, these materials continue to have a high susceptibility to the formation of bacterial plaque.
− The physical properties of Glycolon are slightly worse than Prolene.

According to the results of microbiological examination, the following conclusions were most often identified:

− Rothia mucilaginosa is a Gram-positive, coagulase-negative, encapsulated, non-spore-forming and non-motile coccus that is part of the normal flora of the oropharynx [12]. This bacterium forms biofilms such as Pseudomonas aeruginosa and R. mucilaginosa, a cohabitant in the lower respiratory tract of a patient with bronchiectasis, and is associated with the occurrence of severe bacteremia in immunocompromised patients [13].
− Streptococcus sanguinis, formerly known as Streptococcus sanguis, is a facultative Gram-positive anaerobic bacterium that is part of dental plaque. Streptococcus sanguinis can enter the bloodstream after surgery and colonize the heart valves, especially the mitral and aortic valves, where it is the most common cause of subacute bacterial endocarditis. For this reason, surgeons often prescribe a short course of antibiotics to be taken a few days before and a few days after oral surgery [14].
− Staphylococcus epidermidis is a Gram-positive bacterium that causes biofilm growth and allows other bacteria to bind to an existing one, creating a multi-layered biofilm. Patients with weakened immune systems are at risk of developing infection [15].

According to the results of microbiological research, we found that:

− Monofilament materials are more preferable than polyfilament ones.
− Vicryl most often had the largest number of bacteria on its surface.
− No differences were found between Prolene and Glycolon in intraoperative fences.
− The number of microorganisms on Prolen and Glycolon on day 7 was less than on PGA and Vicryl, while in the last experiment for the detection of Staphylococcus epidermidis, Prolene was better.

Monofilament materials have shown more successful results in comparison with polyfilament materials. In this case, the material of choice for all three studies can be identified as Prolene. The material with the worst performance was Vicryl. It has the highest risk of developing postoperative complications due to its structure.

An article by Pleshkov V.V. was published in the Smolensk Medical Almanac [16]. The sutures used in the study differed from the sutures in this article in several ways. The knot force was measured by means of a hydrodynamic setup, assembled manually by the author of the article. Due to these differences, there are differences in absolute values in Newtons between the experimental results. However, the correlation of measurements within groups in our study and in the experiment of the mentioned article coincides, which allows us to conclude that our study is correct.

Bontsevich D.N. and Kaplan M.L., in their article ‘Physical properties of the suture material influencing the development of septic complications’, in the journal ‘‘Problems of Health and Ecology’’, performed a comparative analysis of the traditional suture materials and surgical threads coated with poly-para-xylylene coating. The research methodology does not differ from the methodology described in our article, only the time range of immersion of the threads in the dyeing medium differs, covering a shorter period. We decided to leave the material in the medium for a longer period to reveal the maximum possible capillarity due to the high residence time of the threads in the tissues of the body [8].

A. S. Knyazyuk, D. N. Bontsevich, N. I. Shevchenko, in the journal “Problems of Health and Ecology” [17], studied the antibacterial properties of the following materials: Caprogent, Lintex, Tveran-CC-Kkr, Nikant and Vikril Plus. These materials are distinguished by their inclusion of antibacterial drugs, which is why these threads initially have a greater microbiological resistance in comparison with the suture materials considered in our article. Now, there are a large number of surgical sutures on the market that do not contain antiseptic solutions in their structure, including Vicryl, Prolene, Glycolon and PGA Resorba, which means we can confirm the relevance of microbiological research within the groups of these materials and comparative analysis of them.

The limitation of our studies includes a small number of samples of each type. Future research will focus on increasing the number of experimental samples, with adjustments to the findings after they have been studied.

## 5. Conclusions

Of the studied suture materials, Prolene had the best microbiological resistance and good mechanical properties. Vicryl sutures showed the worst results in terms of microbiological resistance. These findings could be used for clinical dental practice for cases in which suture materials are used, but require further observations for more evidence-based conclusions.

## Figures and Tables

**Table 1:** Suture materials.

Suture Material	Vicryl	Prolene	Glycolon	PGA Resorba
Production material	Synthetic	Synthetic	Synthetic	Synthetic
Biodegradation	Absorbable	Non-absorbable	Absorbable	Absorbable
Structure	Polyfilament	Monofilament	Monofilament	Polyfilament
Manufacturer	Ethicon	Ethicon	Resorba	Resorba
Country	USA	USA	Germany	Germany

**Table 2:** Results of the study of suture materials mechanical properties.

Mechanical Characteristics	Vicryl Mean + SD Median Min–Max	PGA Mean + SD Median Min–Max	Prolene Mean + SD Median Min–Max	Glycolon Mean + SD Media Min–Max	*p*
Knot strength (in Newtons)	9.79 ± 2.62 9.28 7.2–13.4	7.25 ± 1.41 6.9 5.8–9.1	5.87 ± 0.94 5.75 4.7–7.4	3.52 ± 0.57 3.45 2.9–4.5	*p* = 0.0048
Elongation (in mm)	25.67 ± 3. 56 25.5 21.0–3.0	32.83 ± 3. 06 32.5 29. 0–38.0	34.0 ± 6.1 34.0 26.0–44.0	70.33 ± 17.5 72.5 47.0–95.0	*p* = 0.00049
Capillarity (in mm)	33.33 ± 6.85 30.5 27.5–42.0	27.33 ± 2.25 27.00 25.0–30.0	0.67 ± 0.26 0.5 0.5–1.0	1.33 ± 0.5 2 1.0 1.0–2.0	*p* = 0.00018

**Table 3:** The number of bacteria absorbed on the surface of the threads during the operation.

Bacteria	Vicryl Mean + SD Median Min–Max	PGA Mean + SD Median Min–Max	Prolene Mean + SD Median Min–Max	Glycolon Mean + SD Median Min–Max	*p*
Rothia mucilaginosa (cfu/smear)	140 ± 124 140 20–400	133 ± 77 140 20–240	95 ± 40 100 30–140	68 ± 32 65 20–120	*p* = 0.10664
Streptococcus sanguinis (cfu/smear)	300 ± 102 300 200–500	193 ± 79 200 100–300	79 ± 41 100 25–120	93 ± 53 100 20–150	*p* = 0.00077
Staphylococcus epidermidis (cfu/smear)	134 ± 116 120 20–360	123 ± 96 100 20–300	134 ± 115 100 20–340	184 ± 162 120 40–500	*p* = 0.88753

**Table 4:** The number of bacteria absorbed on the surface of the threads on the 7th day.

Bacteria	Vicryl Mean + SD Median Min–Max	PGA Mean + SD Median Min–Max	Prolene Mean + SD Median Min–Max	Glycolon Mean + SD Median Min–Max	Kruskal–Wallis (*p*)
Rothia mucilaginosa (cfu/smear)	594 ± 130 630 400–730	394 ± 40 395 330–350	283 ± 77 305 120–360	210 ± 96 200 100–380	*p* = 0.0002
Streptococcus sanguinis (cfu/smear)	301 ± 59 300 220–380	269 ± 34 270 220–320	183 ± 39 200 120–220	206 ± 49 220 100–240	*p* = 0.00044
Staphylococcus epidermidis (cfu/smear)	413 ± 101 370 330–600	493 ± 169 500 300–800	214 ± 51 200 160–310	286 ± 66 300 200–360	*p* = 0.00051

## Data Availability

Data are available on request due to the restrictions of the Ethical Committee.
